# Factors Affecting Contrast Sensitivity in Healthy Individuals: A Pilot Study

**DOI:** 10.4274/tjo.93763

**Published:** 2017-04-01

**Authors:** Arzu Seyhan Karatepe, Süheyla Köse, Sait Eğrilmez

**Affiliations:** 1 Kayseri Training and Research Hospital, Ophthalmology Clinic, Kayseri, Turkey; 2 Independent Practitioner, İzmir, Turkey; 3 Ege University Faculty of Medicine, Department of Ophthalmology, İzmir, Turkey

**Keywords:** contrast sensitivity, age, visual function, photopic

## Abstract

**Objectives:**

To determine the demographic and ocular features affecting contrast sensitivity levels in healthy individuals.

**Materials and Methods:**

Seventy-four eyes of 37 subjects (7-65 years old) with refractive errors less than 1.0 diopter, no history of ocular surgery, and 20/20 visual acuity were included in the study. The participants were divided by age into three groups: group 1, 7-19 years, n=11; group 2, 20-49 years, n=15; and group 3, 50-65 years, n=11. All subjects underwent anterior and posterior segment evaluation, intraocular pressure measurements, refraction measurements, and clinical evaluation for strabismus. Contrast static test was performed using Metrovision MonPack 3 vision monitor system after measuring pupil diameter. Photopic and mesopic measurements were taken sequentially from right eyes, left eyes, and both eyes together.

**Results:**

Contrast sensitivity at intermediate and high spatial frequencies was lower with increasing age. Binocular measurements were better than monocular, and mesopic measurements were better than photopic measurements at all spatial frequencies. Contrast sensitivity at higher spatial frequency was lower with hyperopic refraction values.

**Conclusion:**

Increasing age, small pupil diameter, hyperopia, and photopic conditions were associated with lower contrast sensitivity in healthy individuals. Binocular contrast sensitivity measurements were better than monocular contrast sensitivity measurements in all conditions and spatial frequencies.

## INTRODUCTION

Contrast sensitivity measurement is one of the primary methods currently used to evaluate visual function. The eye is able to perceive an object by comparing differences in light level between the target and the background.

Contrast sensitivity is defined as the ability to detect the lowest lumination difference between an object and the background.^1^ Standard visual acuity measurement is done with high contrast conditions. This does not provide any information about visual performance in many of the various activities we perform in our daily lives, such as driving at night or reading in low light, and a patient’s vision cannot be fully assessed by evaluating visual acuity alone.^2^

Contrast sensitivity is one of the main requisites for good vision and, unlike visual acuity, can be affected by many factors. The increasing application of multifocal contact lenses and intraocular lenses (IOLs) has created a new patient group whose visual quality is affected independently of visual acuity. Visual acuity measurement is not an adequate assessment of visual function in these patients, which increases the need for contrast sensitivity and glare testing. However, in order to discuss pathological levels, we first need to determine contrast sensitivity levels in normal individuals and understand the daily living and environmental conditions affecting these levels.

The aim of this study, performed in the Electrophysiology division of the Ege University Faculty of Medicine, Department of Ophthalmology, was to determine standard values for photopic and mesopic contrast sensitivity at different spatial frequencies in specific age groups. We also evaluated factors which may affect contrast sensitivity such as age, pupil diameter, and lighting conditions.

## MATERIALS AND METHODS

Seventy-four eyes of 37 subjects between 7-65 years of age who attended the Ege University Faculty of Medicine, Department of Ophthalmology for routine check-up were included in the study. All subjects underwent a complete ophthalmologic examination including slit-lamp and 90-diometry (D) lens anterior and posterior segment examination, intraocular pressure measurement by applanation tonometry, refraction measurement by autorefractometry, keratometric measurement, and strabismus examination using Hirschberg and cover tests. Prior to contrast sensitivity testing, pupil diameter was measured in the same lighting conditions. Subjects with no ocular pathology, uncorrected 20/20 vision, autorefractometer values less than 1.0 D, and no history of ocular surgery were included.

Contrast sensitivity test was performed using the Metrovision MonPack 3 Vision monitor system. Contrast sensitivity testing was done first in photopic, then in mesopic conditions. At each light level, monocular tests of the right and left eyes (in that order) were followed by binocular tests. During the test, the parameters of the sinusoidal bar such as lumination, contrast, and spatial frequency are adjusted. Each black and white bar was initially presented at low contrast, and the contrast was automatically increased by the instrument. The point at which the subject first perceived the stripes was recorded. The instrument obtained data at spatial frequencies of 0.5, 1.5, 3.0, 6.0, 12.0, and 24.0 cycles/degree (cpd) and at lumination levels of 0-30 decibels (dB).

Prior to contrast sensitivity testing, pupil diameter was measured at the same light level. For comparisons of monocular and binocular function, measurements from the subjects’ dominant eye (right for all subjects) was included in the analysis; measurements taken from subjects’ other eyes (left) were not included in calculations of monocular values. In the contrast sensitivity test, 0.5-1.5 cpd is defined as low, 3.0-6.0 cpd as intermediate, and 12.0-24.0 cpd as high spatial frequency. The subjects were divided by age into three groups in order to compare contrast sensitivity curves: group 1 included 11 subjects 7-19 years old; group 2 included 15 subjects 20-49 years old; and group 3 included 11 subjects 50-65 years old.

### Statistical Analysis

Numerical relationships between age, refractive error, pupil diameter, and contrast sensitivity levels were analyzed by Pearson’s correlation test; paired comparisons such as contrast sensitivity in light/dark conditions and monocular/binocular were analyzed by dependent-samples t-test.

## RESULTS

**Figure 1:**
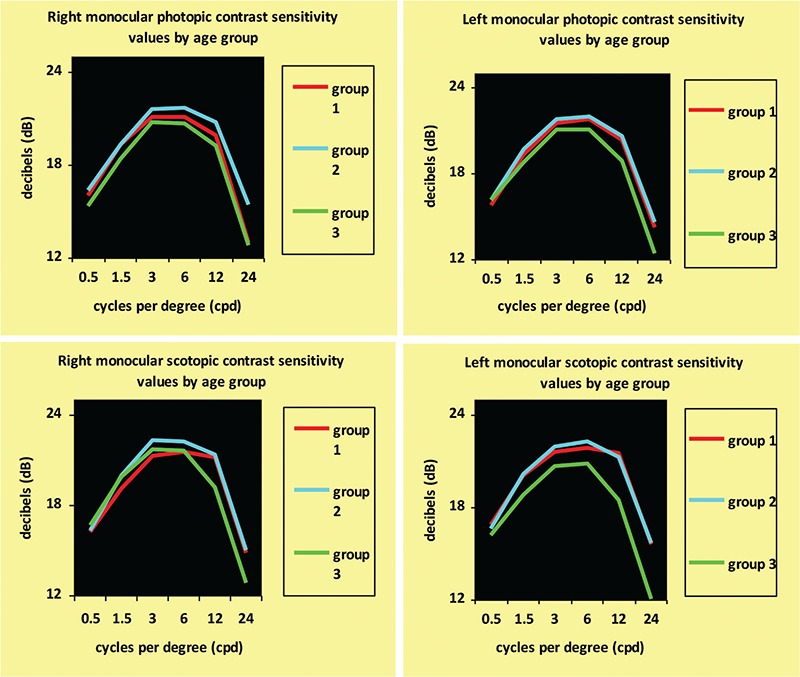
Changes in monocular contrast sensitivity by age group

**Figure 2:**
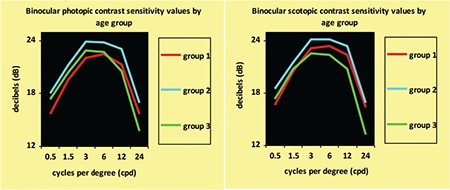
Binocular contrast sensitivity curves by age group

Mean ages of the groups were 11.45±3.55 years for group 1, 35.66±7.62 years for group 2, and 57.09±4.48 years for group 3. Changes between the photopic/mesopic and monocular/binocular contrast sensitivity curves in the age groups are shown in Figure 1 and Figure 2.

**Figure 3:**
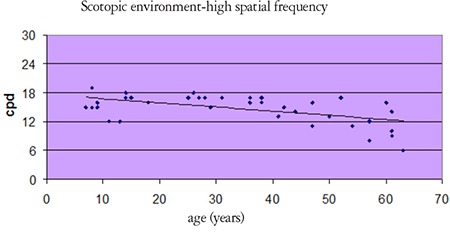
Changes in scotopic contrast sensitivity with age

Statistical analysis revealed no differences between the age groups in contrast sensitivity in photopic conditions, but in mesopic conditions, contrast sensitivity at high spatial frequencies decreased with increasing age (Table 1, Figure 3). Furthermore, with increasing age, pupil diameter measured in both mesopic and photopic conditions was smaller (p<0.01) and refraction tended toward hypermetropia at low refractive errors (p<0.01).

**Figure 4:**
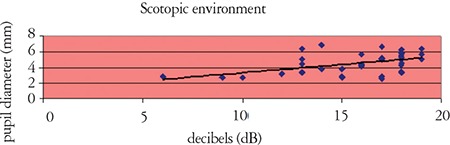
Effect of pupil diameter on scotopic contrast sensitivity values

In photopic conditions, pupil diameter had no effect on contrast sensitivity values. In mesopic conditions, contrast sensitivity values at high spatial frequencies increased in association with larger pupil diameter (Table 2, Figure 4). Evaluation of the association between contrast sensitivity and spherical equivalent at low refractive errors revealed that contrast sensitivity was decreased at intermediate and especially at high spatial frequencies as refraction became hypermetropic (p<0.01). In mesopic conditions, pupil diameter was smaller in hypermetropes (p<0.05).

**Figure 5:**
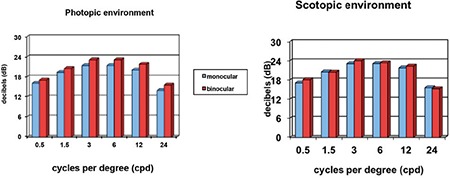
Effect of monocular and binocular measurement on contrast sensitivity values

**Figure 6:**
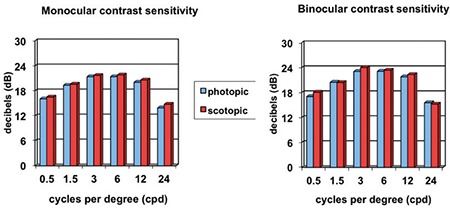
Effect of lighting conditions on contrast sensitivity

In all age groups and at all spatial frequencies, binocular contrast sensitivity values were higher than monocular values (Figure 5), and contrast sensitivity was better in mesopic than photopic conditions (Figure 6). Contrast sensitivity was independent of age and pupil size in photopic conditions.

## DISCUSSION

In recent years, it has become increasingly recognized that visual acuity alone is an inadequate assessment of an individual’s visual quality, and that additional evaluation methods such as contrast sensitivity test are needed.3 Especially with newly developed multifocal intraocular lenses and other refractive procedures, the success of the procedure depends on the contrast sensitivity test results, even if the visual acuity is very good.^4,5,6,7^ Therefore, contrast sensitivity testing is becoming more common in our routine practice.

Histopathologic studies have shown that the macular pigments, photoreceptors, and neural paths are affected in the aging retina.^8,9^ In these studies, it was particularly noted that there is a much larger decrease in the number of rods compared to that of cones.^8,9^ These changes explain the decreases in light sensitivity, contrast sensitivity, and visual acuity as well as prolonged dark adaptation that affect individuals over the age of 50.^8,9^ Some studies have shown that contrast sensitivity does not decrease appreciably with advancing age.^10,11^ However, most studies have reported declines in both photopic and scotopic contrast sensitivity with aging.^12^ It has been proposed that age-related lens sclerosis may play a role in this decrease.^13,14,15^ One of the most comprehensive of these studies is that of Owsley et al.,^16^ which included 91 subjects. They observed decreased contrast sensitivity at high spatial frequencies but found no effect at lower frequencies in subjects over 40 years old; they also noted that small children had high contrast sensitivity at low frequencies, but low sensitivity at intermediate and high frequencies. In a study by Zanglonghi^17^ including 133 eyes, no differences in contrast sensitivity at low spatial frequencies (0.7, 1.4, 2.7 cpd) were observed between age groups spanning a range of 13-82 years old, whereas the 21-30 age group had the highest contrast sensitivity values at high frequencies (5.5, 11, 22 cpd). Arden^18^ and Bradley and Freeman^19^ also showed that the contrast sensitivity levels at low and intermediate frequencies were lower in subjects under 13 years old when compared with the other age groups. In the present study, we found that contrast sensitivity decreased in scotopic conditions and at high spatial frequencies with advancing age, but we found no effect of age on contrast sensitivity in photopic conditions. The contrast sensitivity values of the <20 group were comparable to those of the 20-49 year age group.

Contrast sensitivity is also influenced by pupil size. Changes in pupil size negatively affect contrast sensitivity at both ends of the spectrum. It has been suggested that contrast sensitivity is reduced by diffraction with a miotic pupil, and possibly by spheric aberrations with a dilated pupil.^20^ In our study, we observed no association between pupil diameter and contrast sensitivity other than an increase in contrast sensitivity values at intermediate and high frequencies with pupil dilation in scotopic conditions. Aging is known to bring about yellowing of the lens, as well as reduction in photoreceptor numbers, smaller pupil, and less dilation in low light conditions.^12^ These may have been factors contributing to the reduction in scotopic contrast sensitivity at high frequencies we observed in the older age group in our study.

It is thought that the decrease in contrast sensitivity as refraction moves toward hypermetropia may explain why hypermetropes are more prone to amblyopia than myopes. Controlled studies may elucidate the relationship between contrast sensitivity and amblyopia.

Contrast sensitivity measurements are also influenced by the ambient light level in which the test is performed. It has been reported that contrast sensitivity that is high in photopic conditions decreases in scotopic conditions.^1^ In our study, however, contrast sensitivity measurements were higher in scotopic than photopic conditions. This may be due to an improved ability to distinguish an object from the background as the ambient light darkens. If the background is white and the test object is a dark color, illumination of the background will certainly increase the observer’s ability to recognize the object; however, in this situation, the scotopic environment refers to the ambient light, independent of the background. Increasing the ambient light may decrease contrast sensitivity by creating a counter effect to the illumination of the background.

Our study aimed to evaluate a wide range of ages with the instrument we used, and to compare measurements from school-age children with those of other age groups. For children in particular, explaining the test in detail and extending the duration of the test provided higher test reliability; however, this resulted in there being a limited number of subjects in this age group.

## CONCLUSION

With the astonishingly rapid progression of both medical and surgical therapies, the comparison of newly developed methods with gold standard is inadequate, and contrast sensitivity testing gains importance. It is crucial to create databases of contrast sensitivity values standardized according age, refraction, and pupil diameter. The sample size of our study is insufficient to create such a database. At this stage, we consider this a pilot study which will shed light on future research.

## Figures and Tables

**Table 1:**
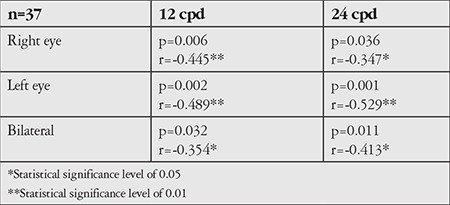
Association between age and contrast sensitivity at high spatial frequencies in scotopic conditions

**Table 2:**
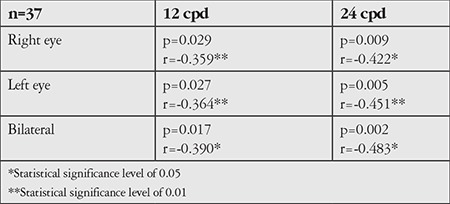
Association between pupil size and contrast sensitivity at high spatial frequencies in scotopic conditions
